# Fiber types and fabric structures influence on weft knitted fabrics

**DOI:** 10.1016/j.heliyon.2022.e09605

**Published:** 2022-06-09

**Authors:** Md. Saiful Hoque, Md. Jakir Hossain, Md. Mahbubur Rahman, Md. Mizanur Rashid

**Affiliations:** a Department of Textile Engineering, Daffodil International University, Bangladesh; b Textile Engineering College, University of Chittagong, Chittagong, Bangladesh

**Keywords:** Weft-knitted fabric, Bursting strength, Wicking tendency, Abrasion-resistant, Pilling resistant

## Abstract

The physical properties of weft knitted fabrics can be modified according to the fabric structure and the raw material used to manufacture the final fabric. This research demonstrates the influence of fiber types and fabric structure on some specific physical properties such as bursting strength, wicking behavior, pilling effect, and abrasion resistance of weft knitted fabrics. For this purpose, in this research study, one natural fiber cotton, one regenerated fiber viscose, and one synthetic fiber polyester were used. At the same time, to avoid any conflicts of the other fabric production factors, the number of feeders, machine diameter, needle gauge, stitch length was kept constant during the production of the weft-knitted fabrics. Moreover, three different structures of single jersey fabric like plain single jersey, single lacoste, and double lacoste were used to produce nine single jerseys of weft knitted fabric, while in each knit structure, three fabrics were produced using 100% cotton, 100% viscose, and 100% polyester fiber. Statistical analysis has been performed along with factorial analysis of variance (ANOVA) followed by simple main effect and simple comparison analysis. The finding illustrates that both fiber types and fabric structure regulate the physical properties of weft knitted fabrics. The polyester fiber seems to possess excellent mechanical properties such as bursting strength, abrasion, and pilling resistance without any influence of fabric structures studied in this research. However, both the fiber types and fabric structure combinedly influence the wicking of weft knitted fabrics. Additionally, it has been assumed that the influence of fiber types and fabric structure on strength, pilling, abrasion-resistant, and wicking properties of fabrics also combined with the areal density and extensibility of weft knitted fabrics.

## 1 Introduction

Clothing is considered the closest environment of the human body constructed from different types of fabrics such as woven, knitted, and non-woven fabrics (Belal, 2018). Among these, weft knitting is a kind of knitted fabric that results from the horizontal interloping of the yarns. These fabric types are getting popular day by day among the consumer as it possesses some excellent mechanical and comfort properties. However, these properties of the fabric highly depend on the type of fibers and fabric structure that have been used to construct the knitted fabric (Farha et al., 2019; Hassan, 2020; M. S. Hoque et al., 2018; S. Hoque et al., 2018; Kalkanci, 2019; Karimian et al., 2013; Oner, 2019; Van Amber et al., 2015).

Among different textile fibers used for manufacturing textile fabrics, either woven or knitted, cotton is the most common (Cook, 1984; Eichhorn et al., 2009; S. Hoque et al., 2018) Cotton is a cellulose-based natural fiber that provides good comfort and desirable physical properties to turn it into a spinnable textile fiber followed by fabrics. However, sometimes it is difficult for the textile manufacturer to get available cotton fiber as high-grade cotton grows well in some specific parts of the world (Jabran and Chauhan, 2019). Therefore, as an alternative, regenerated cellulosic fiber such as *viscose* is getting popular day by day (Chen, 2015). Apart from viscose's easy availability, it also provides excellent drapability, comfort, and minor wrinkle than natural cellulosic fiber like cotton. However, although both natural and regenerated fibers provide good comfort, these fibers lack desirable mechanical strength where the fabrics' optimal mechanical performance is a primary need, such as in sportswear (Eichhorn et al., 2009). Therefore, the use of synthetic fiber such as polyester also prevails widely in the textile manufacturing industry. Polyester fiber not only provides better mechanical performance but also is cheap and readily available.

Along with fiber composition, the properties of weft knitted fabric also largely depend on the type of knit structures used to manufacture the fabric (Choudhary and Ramratan, 2020; Emirhanova and Kavusturan, 2008; Sathish Babu et al., 2020). Weft knitted fabrics can be categorized as single jersey and double jersey (Belal, 2018; Spencer, 2001). The fundamental difference between these two is based on their manufacturing process. One set of knitting beds is used to produce different varieties of single jerseys, whereas two sets of knitted beds are used to produce double jerseys. In general, three types of stitch such as knit stitch, tuck stitch, and miss stitch can be used individually or in a combination to produce different weft knitted fabric structures.

Although both fabric structure and fiber composition affect the fabric properties, there is still a lack of comparative study among fabrics made from 100% natural, regenerated, and synthetic fiber, varying with different weft knitted structures. This study aims to determine how different fiber and knit structures affect the bursting strength, wicking, pilling, and abrasion-resistant properties of weft knitted fabrics. Furthermore, this study carries out a rigorous statistical analytical approach on the bursting strength and wicking properties data generated for this study.

## 2 Literature review

The performance analysis of the knitted fabrics depends on the end-use of the product. However**,** evaluating the fabric's physical properties, such as strength and wicking behavior, is vital while considering the durability and comfort of knitted fabrics. Several researchers also worked to find out how different fibers affect these vital properties. Similarly, researchers investigated the knit fabric structure's influence on different properties of knitted fabrics. Some researchers examined the effect of fourteen different knit structures, including the fabric strength (Emirhanova and Kavusturan, 2008). Through a one-way analysis of variance (ANOVA), they concluded that different knitted fabric structures significantly affect fabrics' strength. The study would have been more focused on the various compositions of fabrics instead of one fabric composition (80% Lambswool-20% Polyamide). Therefore, the result cannot be generalized while considering the other fabric compositions. In another study, the authors looked at the influence of knit structure on knitted fabrics' thermal and moisture management properties (Onofrei et al., 2011). Their study showed that the fabrics' wicking ability is greatly influenced by the types of knitting structures used in that fabric. The authors used different stitch lengths in different knitted structures, which affected the research outcome as stitch length plays a vital role in knit fabric properties (Kane et al., 2007). Another study used six different knitted structures to determine how the fabrics' structural differences affect the fabrics' mechanical and hand properties (Choi and Ashdown, 2000). It found that knit structure combined with tuck and miss stitch produces the best outerwear winter fabrics. However, the limitation in their research, they did not mention the stitch length and other machine parameters used to manufacture the fabrics. Other researchers investigated three derivatives of single jersey fabric made from 100% cotton fiber (Asif et al., 2015). They found that fabric made with the tuck stitch increases the fabrics' areal density, width, pilling resistance, and width shrinkage while decreasing the lengthwise shrinkage and spirality.

In the same way, abrasion resistance is an important property for fabrics, that regulates the quality and efficiency of the process, including pursuance of the product. Numerous studies have been done to measure and analyze factors that contribute to fabrics' abrasion-resistant property. Several factors such as fiber morphology, physical and chemical characteristics, and fabric construction are among the significant factors affecting fabrics' abrasion resistance properties (Jeon et al., 2003; Pastore, Paul Kiekens, 2000). Researchers found that abrasion resistance is higher for the fibers that possess high tensile strength as these fibers have a great capacity to absorb friction under conditions of repeated stretching (Hamburger, 1945; Susich, 1954). Peterson et al. (2021) examined the tenacity of fibers and found that cotton and viscose fiber show almost the same tenacity and force required to break where the polyester fabric has approximately double tenacity and force. In a comprehensive study on abrasion kinetics, they identified the relation of the structural parameter of fibers, yarns, and fabric to abrasion-resistance (Manich et al., 2001). While Backer & Tanenhaus (1951) pointed out the relationship of structural geometry to the abrasion resistance of textile fabrics, the fabric's durability can be significantly altered by modifying the fabric structure without changing the type of fibers used to manufacture that fabric. Another study conducted by Özgüney et al. (2008) using the comparison among several cotton knitted fabrics made of compact and conventional ring yarn also established the powerful effect of fabric construction on abrasion-resistance. However, such studies remain narrow in focus dealing with the combined effect of fiber composition and fabric structure on abrasion resistance properties. Most of the studies either kept fiber composition constant and use different fabric construction or used a similar fabric construction by changing fiber composition. Therefore, further investigation is needed to measure their incorporated impact on fabric properties.

Pilling is another crucial mechanical characteristic of weft knitted fabrics. Pilling formation occurs as bunches or balls of tangled fibers are attached to the fabric by one or more fibers. Many scientists figured out the factors, such as fiber type and length, the number of fiber ends, linear density, cross-sectional shape, yarn twist, hairiness, yarn spinning system, fabric construction, finishing process, that affect pilling (Akaydin and Can, 2010). The pilling problem becomes more acute in the case of synthetic fibers due to their high bending stiffness and circular cross-section (Candan and Önal, 2002). After everything, the structure of the fabric is also critical in determining its susceptibility to pilling. A tight and compact construction exhibits little or no pilling; whereas loosely knitted fabrics have more of a tendency to show pilling (Ukponmwan et al., 1998). To better understand the effect of the knit structures of tuck stitches on pilling resistance, Uyanik & Topalbekiroglu (2017) examined different fabric structures such as single jersey, honeycomb, and Lacoste fabric, but they only used 100% cotton fabric in that study. It has been demonstrated that single jersey fabric has the lowest pilling resistance compared to the fabric having tuck stitches. The fabrics having tuck stitches have much higher porosity**,** weight, and thickness than single jersey fabric, making the fabric more pilling resistant. Another experiment was conducted by Kayseri & Kirtay (2015) to predict the pilling tendency of the cotton interlock knitted fabric through an artificial neural network process. Fabric cover factor and short fiber content have been found as the most significant parameters to influence the pilling tendency of the interlock knitted fabric. In another major study, Candan & Önal (2002) investigated the effect of some ring, and open-end spun cotton yarns, blended yarns (50/50 cotton/polyester, dyed), and fabric variables on the dimensional, pilling, abrasion resistance characteristics of single jersey, Lacoste, and fleece fabric. The results revealed that, unlike plain jersey fabrics, Lacoste fabrics perform very well. In addition, they concluded that, in general, knitted fabrics produced from open-end spun yarns have a lower propensity to pilling. However, further investigation is needed to determine the influence of other fibers on the different knit structures.

In the present study, the impact of fiber composition and fabric structure on bursting strength, wicking behavior, abrasion resistance, and pilling effect has been examined. This study involves the production of nine weft knitted fabrics varying in fabric structure and fiber composition. The machine parameters that can influence fabric properties were kept constant for producing every fabric of this study so that the result remains bias-free from other variables apart from fiber types and fabric structures. Uniquely, this research provides a comparative study on how some mechanical and wicking properties of 100% cotton, 100% viscose, and 100% polyester fiber-based weft knitted fabric change when constructed in different knit structures.

## 3 Materials and methods

This study involves the production of weft-knitted fabrics by controlling the parameters that may affect the properties of knitted fabric regardless of the study's independent variable. The study has been designed as a 3 Χ 3 factorial designs.

Independent Variable:
  Factor A: Fabric Structures (3 levels)
  Factor B: Fiber Types (3 levels)
Dependent Variable:
  Bursting strength, wicking, abrasion and pilling resistant property

### 3.1 Materials

For this study, 100% cotton (34/1 Ne), 100% Viscose (34/1), and 100% Polyester (150 Denier) ring-spun yarns were collected from a spinning mill. Nine different types of single jersey weft knitted fabrics were produced for this study. The information on the fabrics produced for this study is given in Table 1.

**Table 1:**
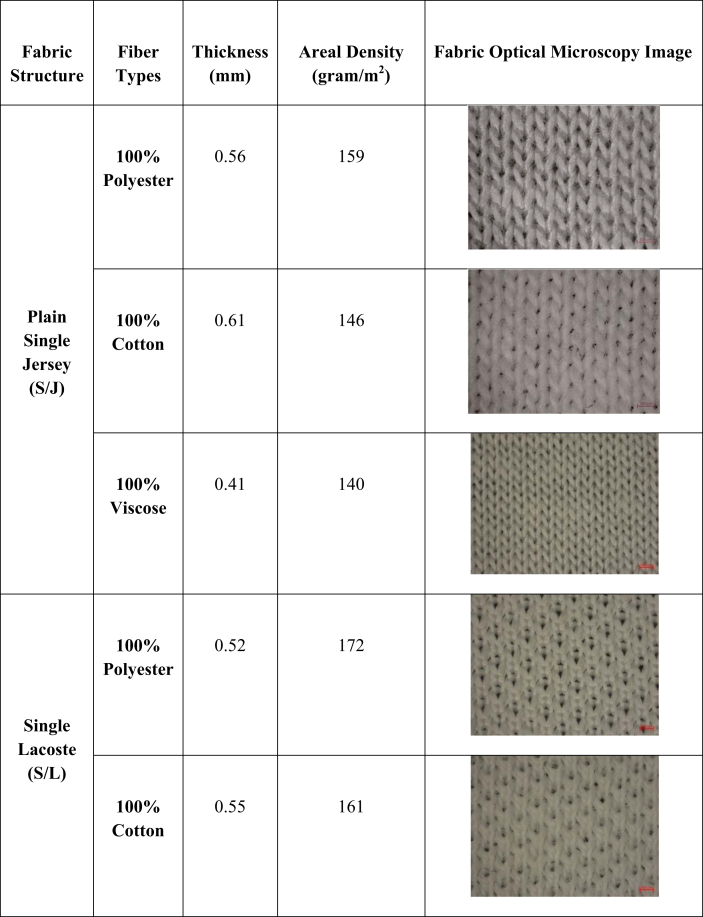

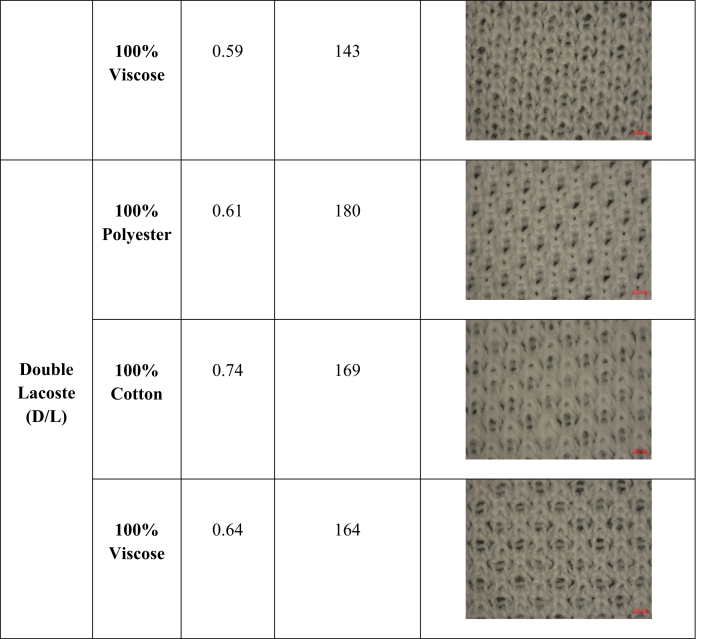
Types of fabric samples produced for this study and its basic specification (mean value). Note: Fabric thickness, and areal density have been measured by following CAN/CGSB-4.2 No. 37–2002, and ASTM D3776-20 standard, respectively (ASTM International, 2020b; CGSB, 2013).

Three different structures from single jersey fabric have been produced to see the fabric structures' effect. Moreover, in each structure, the fabric was produced from three different fibers categorized as natural (cotton), regenerated (viscose), and synthetic (polyester) fibers to determine the effect of fiber types. Table 2 contains the notation diagram, machine cam design, and needle arrangement of different fabric structures prepared for this study. One knitting machine (Pailung Machinery Mill Co. LTD., Model-PL-KS3B/A/C-W, Taiwan) and the same machine parameters were used to produce all the single jersey fabric derivates. Table 3 contains the knitting machine specification used for this study.

**Table 2:**
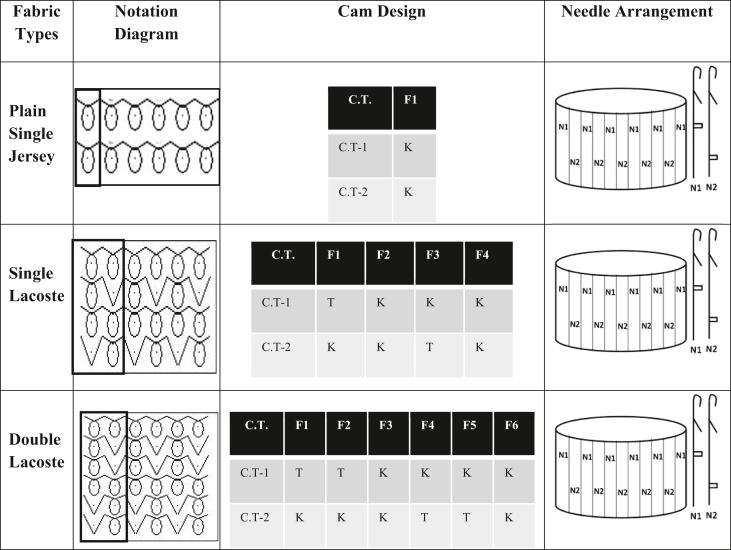
Notation diagram, cam design and needle arrangement of produced fabrics.

**Table 3:** Parameters of knitting machine.

Machine Parameters	Fabric Structure		
	Plain single jersey (S/J)	Single Lacoste (S/L)	Double Lacoste (D/L)
No of Feeders	90	90	90
Machine Diameter (inch)	30	30	30
Machine Gauge (Needle/inch)	24	24	24
Stitch Length (mm)	2.60	2.60	2.60

### 3.2 Conditioning of the specimen

All the fabric samples prepared for this study were conditioned according to ASTM D1776/D1776M-20 (ASTM International, 2020a). Before the characterization, the specimens' condition is essential as high or low humidity can affect the fibers' moisture pick-up equilibrium. By following the standard, for this study, all the specimens were conditioned in a conditioning room for 24 h at 20 ± 2 °C temperature and 65 ± 2% relative humidity room condition.

### 3.3 Measurement of the bursting strength of the fabric

The bursting strength of all produced fabrics was measured following the ISO 13938-2 test standard (ISO, 2019). According to this test method, a circular clamping ring was used to hold the specimen over a diaphragm. Then, the air pressure was applied below the diaphragm, resulting in the distortion of the diaphragm and specimen. For testing the specimens of this study, the pressure was applied over 7.3cm^2^ area of the specimen until the test specimen bursts.

### 3.4 Measurement of wicking of the fabric

Wicking measurement is one of the available techniques for observing the moisture management behavior of fabrics (AATCC, 2012). In this study, vertical wicking test was performed. Following the test standard, distilled water wicking distance at 30 min was measured in lengthwise direction of the fabrics.

### 3.5 Measurement of fabrics’ abrasion resistance

Abrasion resistant test on fabrics were performed in a Martindale abrasion tester according to ISO 12947-2 (ISO, 2016). According to the test method, small circular specimens (about 38 mm in diameter) were inserted into a specimen holder with standard foam behind. The specimen was abraded on a circular area (about 100 mm in diameter) under 9kPa load against a standard wool fabric with a standard felt behind. After that, the test specimens were inspected at predetermined intervals specified in the method and the result is reported as the number of cycles before the end point is reached.

### 3.6 Measurement of fabrics’ pilling resistance

The pilling resistance of all of the fabrics was measured by using an ICI pilling box machine according to ISO 12945-1 (ISO, 2020). During this study, for all the specimens, the machine was set to 60 rpm for 7200 revolutions. After the test, the pilling was visually assessed to provide the rating.

### 3.7 Statistical analysis

For the statistical analysis, this study's data were analyzed using Statistical Package for the Social Sciences (SPSS) 26 software. For the analysis purpose, fabric structure and fiber composition were considered the fixed factors, while mainly factorial analysis of variance (ANOVA) analysis was performed for bursting strength and wicking behavior of fabrics.

## 4 Results and discussions

### 4.1 Effect of fiber and fabric structure on bursting strength of different weft knitted fabrics

The outcome variable that is bursting strength was found to satisfy the assumption of homogeneity of variances based upon the results of Levene's test (F (8, 36) = 0.698, p = .691). A factorial ANOVA was conducted, and the interaction effect was found to be significant (F (4, 36) = 1197.651, p = .000) at p < 0.05 level. The interaction effect yielded an effect size of 0.993, indicating that 99.3% of the fabrics' bursting strength variance was explained by the combined effect of fabric structure and fiber types used to manufacture the fabric.

As the interaction effect was found significant, a simple main effect analysis followed by a simple comparison was performed. For the simple main effect analysis, one independent variable, that is, fabric structure, was held constant at a chosen level, and then the mean differences among all levels of the fiber types (e.g., polyester, cotton, viscose) variable were examined. The simple main effect of fiber types was found significant at every level of fabric structure (Table 4).

**Table 4:** Source table for simple main effect analysis followed by simple comparison for dependent variable, bursting strength.

Source	SS	Df	MS	F	η^2^
**A: Fabric Structure**	5773.333	2	2886.667	44.948	.714
**B: Fiber Types**	1860973.333	2	930486.667	14488.547	.999
**AB**	307663.333	4	76915.833	1197.651	.993
**B at a**_**1**_**level**	261963.333	2	130981.667	2526.977	.12
*B comp1 at a*_*1*_*level*	83740.833	1	83740.833	1303.93	.038
*B comp2 at a*_*1*_*level*	178222.5	1	178222.5	2775.1	.081
**B at a**_**2**_**level**	810930	2	405465	5933.634	.372
*B comp1 at a*_*2*_*level*	670507.5	1	670507.5	10440.46	.308
*B comp2 at a*_*2*_*level*	140422.5	1	140422.5	2186.517	.064
**B at a**_**3**_**level**	1095743.33	2	547871.667	7556.851	.503
*B comp1 at a*_*3*_*level*	901333.333	1	901333.333	14034.651	.414
*B comp2 at a*_*3*_*level*	50410	1	50410	784.934	.023
**S/AB**	2312.000	36	64.222		
**Total**	2176721.996	44			

Note: Comp1- Comparison between Polyester Vs (Cotton + Viscose); Comp2- Comparison between Cotton Vs Viscose. Level a_1_- Plain S/J, level a_2_- S/L and level a_3_- D/L.

The simple main effect was found significant; therefore, a simple comparison was also performed. At every level of fabric structure, two simple comparisons were performed. The first comparison was made between polyester fiber-based fabrics with cotton and viscose fiber-based fabrics. This comparison design was chosen to see if there is a difference between synthetic and cellulosic fiber-based fabrics' bursting strength. The second comparison was performed between cotton and viscose fiber-based fabrics. This comparison was performed to see if there is a difference in fabrics’ bursting strength when made from two kinds of cellulosic fibers, where one is from natural cellulose and the other is regenerated cellulosic fiber. As shown in Table 4, significant differences were observed for all types of comparison.

**Figure 1:**
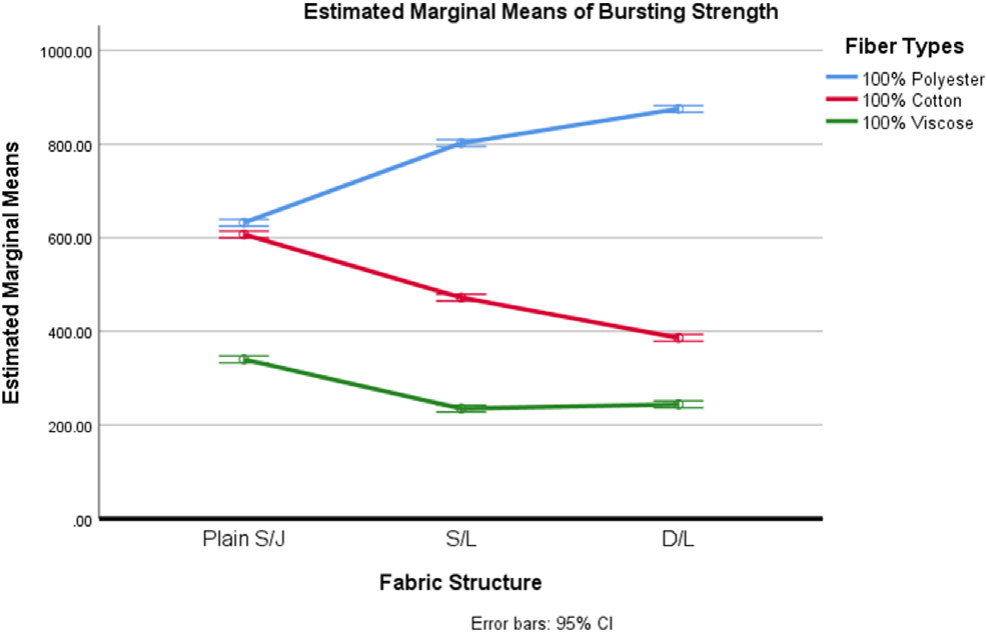
Effect of fabric structure and fiber types on bursting strength of some weft knitted fabrics.

According to Figure 1, 100% polyester-based fabrics exhibited the highest bursting strength**;** whereas 100% viscose-based fabric exhibited less strength in the case of all fabric structures studied in this research. These findings resonances with the basic criteria of polyester fibers. Polyester fibers usually contain a higher crystalline region in their structure than cotton and viscose fibers, which is perhaps attributed to observing the higher strength in 100% polyester fiber-based fabrics (Morton WE, 2008). The 100% viscose fiber-based fabrics exhibited less strength than 100% cotton fiber-based fabrics. This finding may attribute to the lower degree of polymerization value in the viscose fibers than in cotton fibers. In terms of the effect of fabric structure, the bursting strength resulting from S/L and D/L fabric is comparatively less than the plain S/J fabric while the fabric is constructed from either 100% cotton or 100% viscose fibers. Both S/L and D/L fabrics contain tuck stitches that are believed to be responsible for reducing the extensibility of the knitted fabric (Uyanik and Topalbekiroglu, 2017). According to literature, the bursting strength of knitted fabrics reduces if the extensibility of the fabric reduces (Saville, 2000). However, this behavior does not echo the behavior of the 100% polyester fiber-based fabrics examined in this study. Perhaps, comparatively, the higher areal density of polyester-based fabrics (Table 1) neutralized the effect of tuck stitches on the bursting strength and yielded more strength than plain single jersey fabrics. Though in the case of cotton and viscose fiber areal density increased, seems due to the fiber characteristics they cannot neutralize the effect of tuck stitches on the bursting strength.

### 4.2 Effect of fiber and fabric structure on moisture management (wicking) of different weft knitted fabrics

The assumption of the homogeneity of variance was satisfied based on Levene's test (F (8, 36) = 0.926, p = .507). Factorial ANOVA analysis data of the fabrics revealed a significant interaction effect of fabric structure and fiber types on the wicking behavior of fabrics (F (4, 36) = 49.791, p = .000) at p < 0.05 level. The effect size of 0.847 was found, which indicates that 84.75% of the variances in the fabrics' lengthwise wicking were explained by the combined effect of fabric structure and fiber types used to manufacture the fabric.

As the interaction effect was found significant, a simple main effect followed by a simple comparison was performed (Table 5). Simple main effect analysis was performed in each fabric structure for all the levels of fiber types. For each structure, the simple main effects of fiber were found significant, as shown in Table 5. Then, two simple comparisons were performed for each simple main effect analysis. Unlike the bursting strength, it has been found that there are no significant differences in the wicking behavior between cotton and viscose fiber-based plain S/J fabrics. However, other simple comparisons were found significant, as shown in Table 5.

**Table 5:** Source table for simple main effect analysis followed by simple comparison for dependent variable, Wicking behavior of fabrics.

Source	SS	Df	MS	F	η^2^
**A: Fabric Structure**	116.744	2	58.372	109.791	.859
**B: Fiber Types**	113.078	2	56.539	106.343	.855
**AB**	105.889	4	26.472	49.791	.847
**B at a**_**1**_**level**	22.933	2	11.466	13.49	.065
*B comp1at a*_*1*_*level*	22.53	1	22.53	42.35	.063
*B comp2 at a*_*1*_*level*	0.4	1	0.4	**0.751**	.001
**B at a**_**2**_**level**	128.133	2	64.066	238.75	.361
*B comp1at a*_*2*_*level*	32.033	1	32.033	60.21	.09
*B comp2 at a*_*2*_*level*	96.1	1	96.1	180.64	.27
**B at a**_**3**_**level**	67.9	2	33.95	71.223	.191
*B comp1at a*_*3*_*level*	12.675	1	12.675	23.83	.035
*B comp2at a*_*3*_*level*	55.225	1	55.225	103.81	.155
**S/AB**	19.140	36	0.532		
**Total**	354.851	44			

Note: Comp1- Comparison between Polyester Vs Cotton + Viscose; Comp2- Comparison between Cotton Vs Viscose. Level a_1_- Plain S/J, level a_2_- S/L and level a_3_- D/L.

**Figure 2:**
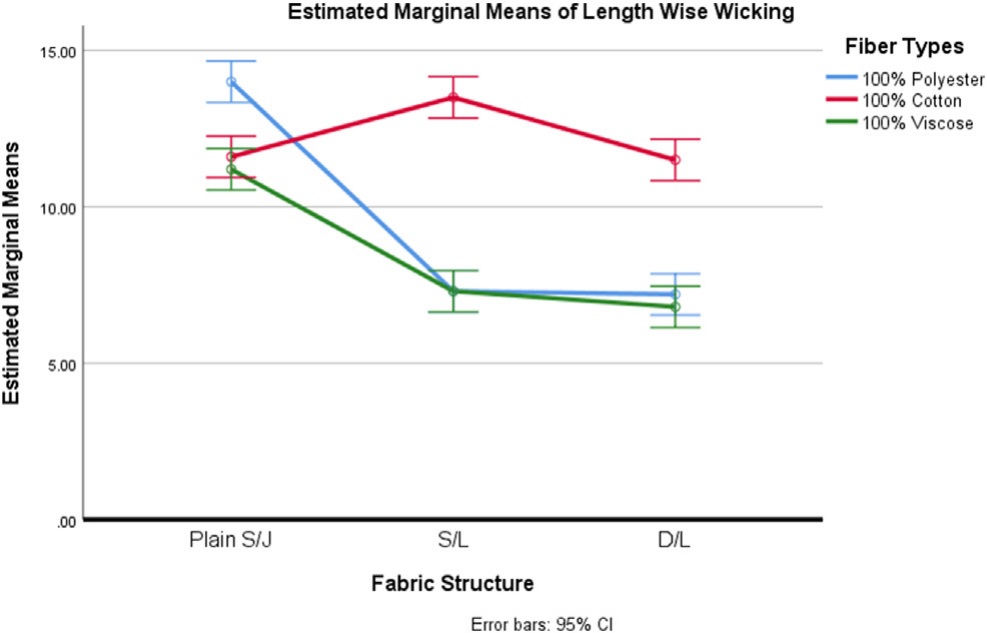
Effect of fabric structure and fiber types on wicking of some weft knitted fabrics.

As shown in Figure 2, for the plain single jersey fabrics, the wicking tendency of cotton and the viscose-based fabric is quite similar. This behavior completely makes sense as cotton and viscose both are cellulosic fibers that contains a hydroxyl group, and this hydroxyl group can easily facilitate the movement of water molecules in the fiber (Morton and Hearle, 2008). Also, both cotton and viscose fiber contain a decent portion of the amorphous region which allows water molecules to penetrate into the fiber structure (Hossain et al., 2020; Rashid et al., 2020). On the contrary, polyester-based fabric has a higher wicking tendency than these. This is because liquid passes across yarns and fabrics by capillary forces, and these capillary forces draw water into the capillary spaces to wet the fiber. The polyester fibers may contain less hairiness than the cotton and viscose fibers; therefore, perhaps this less hairiness allows the polyester fiber to form fine capillary channels that can be attributed to the high wicking tendency for the polyester fiber-based plain S/J fabrics.

However, this behavior of fibers had changed when we considered S/L and D/L fabrics. The S/L and D/L fabrics are denser because of having tuck stitches. Then, areal density of the fabric along with moisture regain of the fiber probably played a role. Both S/L and D/L cotton-based fabrics showed higher areal density (Table 1), and likely the effect of areal density was more in the wicking than the fine capillary channel as found in the literature (Yang et al., 2021). But, both S/L and D/L viscose-based fabric having higher moisture regain and almost similar areal density compared to cotton fiber showed lower wicking behavior. Maybe using finer count yarn has been partly responsible as finer count viscose yarn presents discontinuous capillaries and the tortuous path the liquid has to take (Banu et al., 2013). At the same time, the polyester-based fabric had higher areal density but lower thickness than cotton-based fabrics as synthetic fiber like polyester has higher fiber orientation including the more intimate the contact between fibers. On the other hand, cellulose fiber such as cotton and viscose fiber has different labels of orientation, this most likely leads cotton and viscose-based fabric to become low areal density (Mogahzy, 2008). Moreover, polyester fiber has very low moisture regain, which hindered the wicking while considering the dense polyester-based fabrics like S/L and D/L with low pore channel (Das et al., 2009).

However, as per the statistical analysis, we did not find any statistically significant effect of areal density (p = 0.391) on the wicking behavior of the weft knitted fabrics studied in this research.

### 4.3 Effect of fiber and fabric structure on abrasion resistance of different weft knitted fabrics

As seen in Table 6, there was no thread breakdown after 35,000 cycles in the case of plain single jersey fabric. However, when it comes to lacoste fabrics, both 100% cotton and 100% viscose for single and double lacoste show several thread breakdowns after certain revolutions. This may have happened as Lactose fabric has tuck stitches that negatively affect abrasion behavior, and threads breakdown occurred at the point where tuck stitches are thrown away (Candan and Önal, 2002). On the other hand, the low abrasion resistance of 100% cotton and 100% viscose fabric may be attributed to these fibers' inner structure. In fact, cotton fiber has an intermolecular cross-link in cellulose which reduces the mobility at the polymer chains and makes cotton structure brittle. As the number of cross-linked increases, the abrasion resistance of the fiber decreases (Dhiman and Chakraborty, 2017; Rizwan et al., 2019). Similarly, viscose fibers have the lowest degree of polymerization, the lowest degree of crystallinity, and a high amount of amorphous regions, which may have contributed to the poor abrasion-resistant property of viscose-based weft knitted fabrics (Basit et al., 2018; Stana-kleinschek et al., 2003). Fabrics’ tensile property also confounded with the abrasion-resistant property of weft knitted fabrics. Cotton and viscose present almost the same low tensile strength, which may be the probable reason to show several thread breakdowns of those fibers during the abrasion test. In contrast, polyester fabric significantly shows more abrasion resistance as it has good tensile strength compared to natural fiber (Koç and Ç in ç ik, 2013).

**Table 6:**
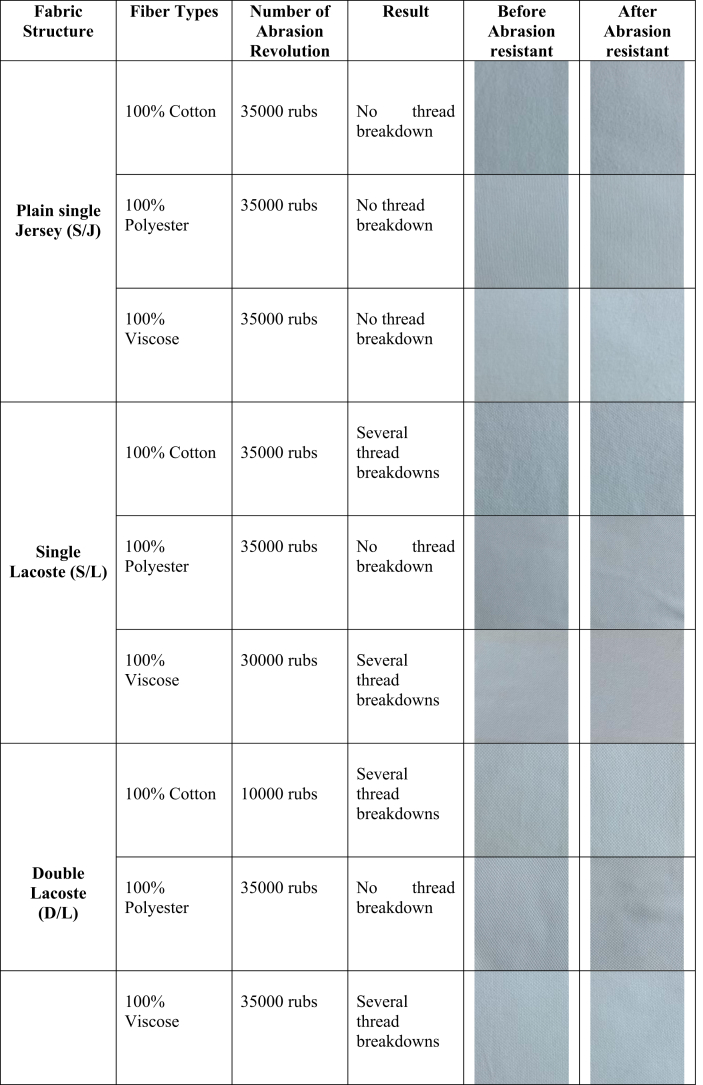
Effect of fabric structure and fiber types on thread breakdown of the tested specimens with abrasion cycles.

### 4.4 Effect of fiber and fabric structure on pilling properties of different weft knitted fabrics

**Figure-3:**
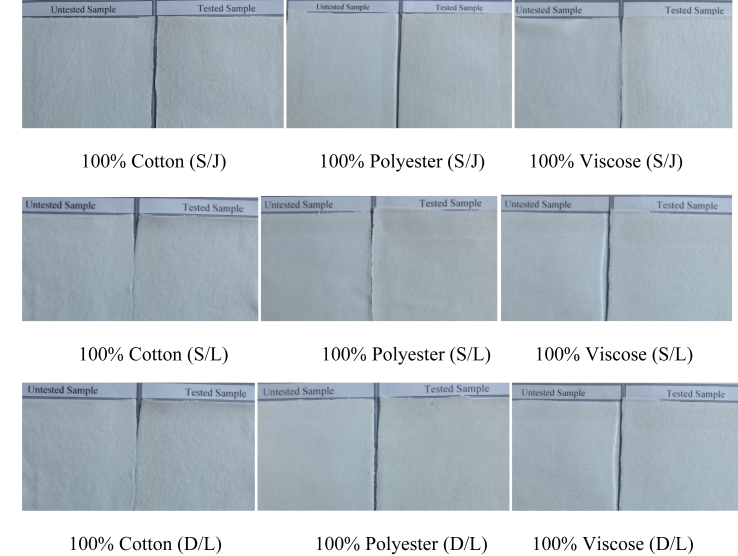
Picture of untested and tested sample during pilling test.

Pilling ratings of tested fabrics shown in Table 7 revealed that for 100% cotton (Plain and Single Lacoste) fabric, pilling grade is comparatively less than that of other fabrics. The most likely cause may be the hairiness of cotton fiber that holds some projecting fibers. And, this short-staple length leads to a lower pilling rating than other fabrics (Kayseri and Kirtay, 2015). By contrast, viscose and polyester fibers-based fabrics showed the same pilling resistance for all types of weft knitted fabric structures studied in this research. Viscose fiber possesses comparatively lower fiber strength that may allow viscose fibers to leave the fabric surface more easily, resulting in fewer pills remaining on the fabric surface at the end of the tests and, consequently, exhibiting better pilling grade than cotton fiber-based fabrics (Göktepe, 2002). In relation to polyester, it is a filament fiber that has less hairiness and cannot generate many microfibers during abrasion. This feature may contribute to polyester showing better pilling resistant properties than cotton fiber-based fabrics (Zambrano et al., 2019). The visual representation in Figure 3 also strengthens this explanation, there has no significant impact of pilling on 100% polyester and 100% viscose-based fabric. Apart from that, the result of pilling resistance of this study concludes that the lacoste knit structure, either single lacoste or double lacoste, has a higher resistance to pilling than the plain single jersey structure. Several factors are known to affect the pilling rating, among them, the promising explanation may be that the rate of pill wear-off in lacoste fabric is higher than pill formation. It is also possible that the number of the cycle (7200, 60 rpm) was not long enough for the completion of pill formation in these fabrics (Paek, 1989).

**Table 7:** Effect of fabric structure and fiber types on Pilling Grade of the tested samples.

Fabric Structure	Plain single Jersey (S/J)			Single Lacoste (S/L)			Double Lacoste (D/L)		
Fiber Types	100% Cotton	100% Polyester	100% Viscose	100% Cotton	100% Polyester	100% Viscose	100% Cotton	100% Polyester	100% Viscose
Pilling Grade	4	4–5	4–5	4	4–5	4–5	4–5	4–5	4–5

## 5 Conclusion

The present study was designed to determine the effect of the bursting strength, wicking behavior, pilling effect, and abrasion-resistance of some commercially in demand weft knitted fabrics based on fabric structure and fiber used to manufacture the final fabric.

• The most obvious finding to emerge from this study is that polyester-based fabrics' bursting strength is more than cotton and viscose-based fabrics. Also, fabric structure especially S/L and D/L fabric have a great influence on the bursting strength of both cotton and viscose-based fabric but no impact on polyester fabric.
• Plain S/J fabric made of all three types of fiber (cotton, polyester, and viscose) shows the highest wicking tendency whereas S/L and D/L fabric shows downward tendency except 100% cotton S/L fabric. Porous fabric structure and fiber characteristic seem to affect the wicking behavior positively.
• Fabric produced from 100% cotton fiber has less pilling resistance than polyester and viscose-based fabric. In addition, tight and compact fabric structure helps to represent excellent pilling grade.
• This research further analyses the abrasion-resistant behavior of the produced weft knitted fabrics. Hundred percent polyester fiber-based fabrics shows the highest abrasion-resistant properties than 100% cotton or 100% viscose fiber-based fabrics. Moreover, due to fabric construction, both S/L and D/L shows higher abrasion resistance.
• This project is the first comprehensive investigation to see the impact of a few physical properties because of changing fabric structure and fiber types on some specific weft knitted fabrics. This study adds to the growing body of research that indicates both fabric structure and fiber types are the essential functions to determine the physical properties of weft knitted fabric. Taken together, this study has raised important questions about the nature of fabric structure and fiber properties that may help the weft knitting industry including academicians to make an informed decision for producing weft knitted fabrics considering the desired fabric properties.

## Declarations

### Author contribution statement

Md. Saiful Hoque: Conceived and designed the experiments; Performed the experiments; Analyzed and interpreted the data; Contributed reagents, materials, analysis tools or data; Wrote the paper.

Md. Mahbubur Rahman: Performed the experiments; Contributed reagents, materials, analysis tools or data; Wrote the paper.

Md. Mizanur Rashid: Performed the experiments; Contributed reagents, materials, analysis tools or data; Wrote the paper.

Md. Jakir Hossain: Contributed reagents, materials, analysis tools or data; Wrote the paper.

### Funding statement

This research did not receive any specific grant from funding agencies in the public, commercial, or not-for-profit sectors.

### Data availability statement

Data will be made available on request.

### Declaration of interests statement

The authors declare no conflict of interest.

### Additional information

No additional information is available for this paper.
